# Visualizing stability: a sensitivity analysis framework for t-SNE embeddings

**DOI:** 10.3389/fbinf.2025.1719516

**Published:** 2026-01-02

**Authors:** Susanne Zabel, Philipp Hennig, Kay Nieselt

**Affiliations:** 1 Institute for Bioinformatics and Medical Informatics, University of Tübingen, Tübingen, Germany; 2 Department of Computer Science, University of Tübingen, Tübingen, Germany

**Keywords:** t-SNE, uncertainty, explainable machine learning, error propagation, visualization, data insights

## Abstract

t-distributed Stochastic Neighbour Embedding (t-SNE) is a cornerstone for visualizing high-dimensional biological data, where each high-dimensional data point is represented as a point in a two-dimensional map. However, this static map provides no information about the stability of the visual layout, the features that influence it, or the impact of uncertainty in the input data. This work introduces a computational framework that allows one to extend the standard t-SNE plot by visual clues about the stability of the t-SNE embedding. First, we perform a sensitivity analysis to determine feature influence: by combining the Implicit Function Theorem with automatic differentiation, our method computes the sensitivity of the embedding w.r.t. the input data, provided in a Jacobian of first-order derivatives. Heatmap-visualizations of this Jacobian or summarizations thereof reveal which input features are most influential in shaping the embedding and identifying regions of structural instability. Second, when input data uncertainty is available, our framework uses this Jacobian to propagate error, probabilistically quantifying the positional uncertainty of each embedded point. This uncertainty is visualized by augmenting the plot with hypothetical outcomes, which display the positional confidence of each point. We apply our framework to three diverse biological datasets (bulk RNA-seq, proteomics, and single-cell transcriptomics), demonstrating its ability to directly link visual patterns to their underlying biological drivers and reveal ambiguities invisible in a standard plot. By providing this principled means to assess the robustness and interpretability of t-SNE visualizations, our work enables more rigorous and informed scientific conclusions in bioinformatics.

## 1 Introduction

At the intersection of artificial intelligence and biology, data visualization serves as a critical bridge between complex computational models and human insight. t-Distributed Stochastic Neighbor Embedding (t-SNE) is a cornerstone of modern data exploration, enabling researchers to visualize the structure of high-dimensional datasets, such as clusters, in intuitive mostly two-dimensional maps (Van der Maaten and Hinton, 2008). Its widespread adoption in bioinformatics is a testament to its power in revealing meaningful patterns and guiding hypothesis generation, from identifying cell populations in single-cell transcriptomics (e.g., Kobak and Berens, 2019; Zhou and Jin, 2020) to visualizing sample relationships in proteomic data (Abdelmoula et al., 2016; Schessner et al., 2022) and genomic data (Platzer, 2013), and many other applications.

Despite its power, a standard t-SNE plot is a static endpoint that obscures critical information. This creates two fundamental challenges for rigorous interpretation. First, the plot provides no direct insight into the feature influence: we cannot easily determine which input features are most responsible for a point’s placement. While linear methods like PCA (Pearson, 1901; Hotelling, 1933) yield feature loadings, the non-linear t-SNE model remains a “black box”. Second, biological data is inherently noisy (Kavran and Clauset, 2021), and standard t-SNE ignores this measurement uncertainty. This leaves a crucial question unanswered: how would the embedding change if we could account for this input noise, and how could this be visually implemented?

Recognizing these and other challenges, an active field of research has emerged to enhance t-SNE’s reliability. One line of work assesses the fidelity of the embedding by quantifying distortion errors or identifying false neighbors, thus evaluating the map’s quality with respect to a fixed input dataset (Han et al., 2022; Zhao et al., 2024; Ozgode Yigin and Saygili, 2022). Other approaches modify the algorithm itself, either by incorporating supervised class labels to improve cluster separation (Meng et al., 2023; Hajderanj et al., 2019) or by integrating input uncertainty directly into the optimization objective, as in the Ut-SNE’s preprint (Ma and Chen, 2024). More recently, gradient-based methods using the Implicit Function Theorem have been introduced for local explanation (Corbugy et al., 2024). However, this initial gradient-based approach relies on a simplifying assumption, analyzing a single point while holding all others fixed. While these are all valuable contributions, a framework is still needed that can (1) analyze the sensitivity of the complete, coupled t-SNE embedding in a post-hoc manner, and (2) use this analysis to address the critical and distinct problem of propagating input data uncertainty.

Here, we introduce such a framework. It is crucial to distinguish the stability we address here from the well-known variability of t-SNE arising from different random initializations or hyperparameter choices (Belkina et al., 2019). Such studies concern the algorithm’s global stability. Our work, in contrast, focuses on the local stability of a single, converged t-SNE solution. We ask: given a specific, optimized embedding, how robust is it to small perturbations in the input data, what drives this sensitivity, and how can this be visually integrated into the plot? To answer these questions, our central contribution is a method based on the Implicit Function Theorem (IFT) (Cauchy, 1831; Krantz and Parks, 2002) to efficiently compute the complete sensitivity Jacobian of the embedding, overcoming the intractability of differentiating through the optimizer. This approach extends the concept of uncertainty-aware dimensionality reduction we previously developed for PCA (Zabel et al., 2023), applying it to the more complex setting of t-SNE. The resulting Jacobian enables two key visual enhancements—feature influence heatmaps and uncertainty visualizations—that provide a practical toolkit for moving beyond static plots towards a more robust and interpretable use of t-SNE.

We first detail the theory behind our IFT-based approach followed by our visualization design decisions, and then apply our framework to a biological time-series dataset, demonstrating how sensitivity analysis can deconstruct the embedding to identify key biological drivers and how uncertainty visualization can reveal structural ambiguities. Our work provides a practical toolkit for moving beyond static plots towards a more interpretable use of t-SNE.

## 2 Methods

Our framework aims to conduct a sensitivity analysis of a t-SNE embedding to its input data. This sensitivity, which is quantified by a Jacobian matrix, then enables both direct feature influence analysis and principled uncertainty propagation. A principled uncertainty propagation determines how uncertainties in input measurements affect the uncertainty of a calculated quantity (in our case the embedded values). For an efficient calculation of the Jacobian, we will leverage the Implicit Function Theorem.

### 2.1 t-Distributed Stochastic Neighbor Embedding (t-SNE)

t-Distributed Stochastic Neighbor Embedding (t-SNE) (Van der Maaten and Hinton, 2008) is a nonlinear dimensionality reduction technique that computes a low-dimensional embedding of high-dimensional data while preserving local neighborhoods. Given a set of high-dimensional data points $Y=\left\{y_{1},\ldots ,y_{N}\right\}$ , where each $y_{i}$ is of dimension D , the algorithm first converts the Euclidean distances between points into joint probabilities, $p_{ij}$ , representing their pairwise similarity. To compute these probabilities, for each pair (i,j) we first compute the conditional probability that $y_{j}$ is a neighbor of $y_{i}$ :

$$p_{j|i}=\frac{\exp\left(-\frac{\|y_{i}-y_{j}{\|}^{2}}{2\sigma_{i}^{2}}\right)}{\underset{k\neq i}{\sum }\exp\left(-\frac{\|y_{i}-y_{k}{\|}^{2}}{2\sigma_{i}^{2}}\right)}$$ (1)

The variance $\sigma_{i}^{2}$ is chosen on a per-point basis to match a user-defined hyperparameter known as perplexity, which controls neighborhood size. The probability $p_{ij}$ is symmetrized by

$$p_{ij}=\frac{p_{j|i}+p_{i|j}}{2N}.$$ (2)

For the corresponding low-dimensional embedding points $Z=\left\{z_{1},\ldots ,z_{N}\right\}$ , where each $z_{i}$ is typically of dimension P=2 , a similar set of joint probabilities, $q_{ij}$ , is computed. A key feature of t-SNE is its use of a heavy-tailed Student’s t-distribution with one degree of freedom for this low-dimensional space, which helps to alleviate the crowding of points:

$$q_{ij}=\frac{{\left(1+\|z_{i}-z_{j}{\|}^{2}\right)}^{-1}}{\sum_{k\neq l}{\left(1+\|z_{k}-z_{l}{\|}^{2}\right)}^{-1}}.$$ (3)

The goal of t-SNE is to find an embedding Z where the probability distribution $Q=\left\{q_{ij}\right\}$ best models the distribution $P=\left\{p_{ij}\right\}$ . This is achieved by minimizing the Kullback-Leibler (KL) divergence between the two distributions. The objective is thus to minimize the cost function C , which can be written as a function of the vectorized input data y and the embedding z :

$$C\left(y,z\right)=\underset{i\neq j}{\sum }p_{ij}\left(y\right)\log\frac{p_{ij}\left(y\right)}{q_{ij}\left(z\right)}.$$ (4)

This cost function is minimized using an iterative optimization method, typically gradient descent, to find the final, optimal embedding z* . The gradient of C w.r.t. $z_{i}$ is

$$\frac{\partial C}{\partial z_{i}}=4\underset{j\neq i}{\sum }\left(p_{ij}-q_{ij}\right)\;\frac{\left(z_{i}-z_{j}\right)}{1+\|z_{i}-z_{j}{\|}^{2}}.$$ (5)

### 2.2 Sensitivity analysis: problem formulation

To analyze the sensitivity of the t-SNE embedding, we must first formalize the relationship between the input and its output. Let the high-dimensional input data be a matrix $Y\in R^{N\times D}$ and its vectorized form be $y=\text{vec }\left(Y\right)\in R^{ND}$ . The t-SNE algorithm operates on a specific point estimate of this data, which we denote as the mean y* . Through the optimization described in Section 2.1, it produces a corresponding optimal low-dimensional embedding, denoted $z*\in R^{NP}$ (where P is typically 2).

Our primary goal is to perform a sensitivity analysis by computing the Jacobian of the optimal solution map, $\frac{\partial z*\left(y\right)}{\partial y}$ . This matrix quantifies how the final, optimized embedding z* changes in response to perturbations of the input data y . A naïve approach to compute this Jacobian would be to apply automatic differentiation (AD) through the entire iterative gradient descent procedure. However, a typical t-SNE optimization involves hundreds or thousands of steps. Unrolling this entire process creates an exceptionally large computational graph, leading to prohibitive memory consumption and potential numerical instability. Therefore, a more direct and memory-efficient method is required. Instead of differentiating through the optimizer, our approach is to differentiate the optimality conditions at the final converged solution.

### 2.3 Computing embedding sensitivities via the implicit function theorem

Our method relies on the stationary point condition of the t-SNE optimizer. At a converged (local) minimum (y*,z*) , the gradient of the cost function with respect to the embedding parameters (Equation 5) is zero:

$$\nabla_{z}C\left(y^{*},z^{*}\right)=0.$$ (6)

We can define a function $G\left(y,z\right)=\nabla_{z}C\left(y,z\right)$ . The condition in Equation 6 is thus G(y*,z*)=0 , which implicitly defines the optimal embedding z* as a function of the input y . The Implicit Function Theorem formalizes this relationship.

The Implicit Function Theorem (Cauchy, 1831) states that for a continuously differentiable function $G:\;R^{n}\times R^{m}\to R^{m}$ , if $\left(x*,y*\right)\in R^{n}\times R^{m}$ is a point such that G(x*,y*)=0 and the Jacobian matrix $\frac{\partial G}{\partial y}$ is invertible at (x*,y*) , then there exists a continuously differentiable function $f:\;R^{n}\to R^{m}$ in a neighborhood of x* such that G(x,f(x))=0 . The Jacobian of this implicit function is given by:

$$\frac{\partial f\left(x\right)}{\partial x}=-{\left[\frac{\partial G\left(x,y\right)}{\partial y}\right]}^{-1}\frac{\partial G\left(x,y\right)}{\partial x}.$$ (7)

To apply this theorem to our problem, we map our variables: $x\to y\in R^{ND}$ , $y\to z\in R^{NP}$ , and $G\left(y,z\right)\to \nabla_{z}C\left(y,z\right)$ . The required Jacobians of G are the second-order derivatives of the original cost function C :

-
$\frac{\partial G}{\partial z}=\frac{\partial }{\partial z}\left(\nabla_{z}C\right)=\frac{\partial^{2}C}{\partial z^{2}}$ (the Hessian with respect to z ).
-
$\frac{\partial G}{\partial y}=\frac{\partial }{\partial y}\left(\nabla_{z}C\right)=\frac{\partial^{2}C}{\partial y\partial z}$ (the mixed-partial derivatives).

Substituting these into the IFT (Equation 7) yields the Jacobian of the optimal embedding map z*(y) :

$$\frac{\partial z^{*}\left(y\right)}{\partial y}=-{\left[\frac{\partial^{2}C\left(y,z^{*}\right)}{\partial z^{2}}\right]}^{-1}\frac{\partial^{2}C\left(y,z^{*}\right)}{\partial y\partial z}.$$ (8)

However, a critical challenge arises: the Hessian matrix $H_{zz}=\frac{\partial^{2}C}{\partial z^{2}}$ , whose inverse is required by the theorem (Equation 8), is singular for the standard t-SNE cost function. The singularity stems from the fact that the t-SNE cost depends only on pairwise Euclidean distances between embedded points. Consequently, the cost function is invariant to transformations that preserve these distances—namely, global translations and rotations of the entire embedding. This invariance means the Hessian has a null space of dimension three and is therefore not invertible.

Since the absolute position and orientation of a t-SNE plot are irrelevant for interpretation, we employ the Moore-Penrose pseudoinverse of the Hessian $\left(H_{zz}^{+}\right)$ to satisfy the IFT’s requirement on the subspace of meaningful variation. The pseudoinverse inverts the transformation on the subspace orthogonal to the null space while mapping the invariant directions to zero. This yields our final expression for the sensitivity Jacobian:

$$\frac{\partial z^{*}\left(y\right)}{\partial y}=-{\left[\frac{\partial^{2}C\left(y,z^{*}\right)}{\partial z^{2}}\right]}^{+}\frac{\partial^{2}C\left(y,z^{*}\right)}{\partial y\partial z}.$$ (9)

This approach allows us to compute the complete sensitivity profile of a t-SNE embedding using only derivatives of its cost function evaluated at the single converged solution point.

### 2.4 Approximate Gaussian error propagation through t-SNE

With the sensitivity Jacobian computed, we can perform principled uncertainty propagation as a direct application. Assuming the input uncertainty is modeled as a Gaussian distribution, $p\left(y\right)=N\left(y;y*,\Sigma_{y}\right)$ , we can approximate the output distribution using a first-order Taylor expansion of the solution map z*(y) around y* :

$$z^{*}\left(y\right)\approx z^{*}\left(y^{*}\right)+{\left.\frac{\partial z^{*}\left(y\right)}{\partial y}\right|}_{y^{*}}\left(y-y^{*}\right).$$ (10)

Under this linear approximation, the output distribution is also approximately Gaussian, $p\left(z*\right)\approx N\left(z*;z*\left(y*\right),\Sigma_{z}\right)$ , with a covariance matrix $\Sigma_{z}\in R^{NP\times NP}$ given by the rules of Gaussian error propagation:

$$\Sigma_{z}\approx \left({\left.\frac{\partial z^{*}\left(y\right)}{\partial y}\right|}_{y^{*}}\right)\Sigma_{y}{\left({\left.\frac{\partial z^{*}\left(y\right)}{\partial y}\right|}_{y^{*}}\right)}^{⊺}.$$ (11)

### 2.5 Design of visualization

**FIGURE 1:**
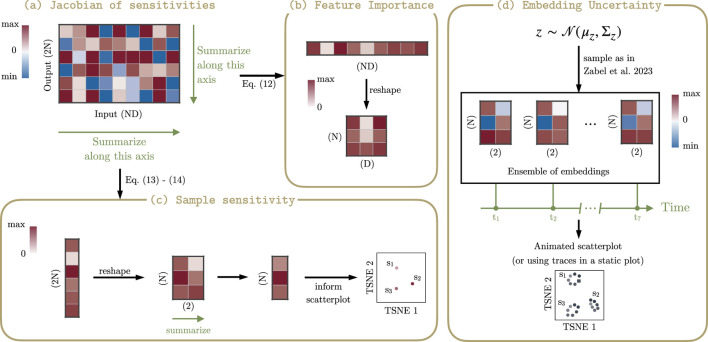
Schematic Overview of the Proposed Visualization Framework. Our method uses the sensitivity Jacobian as a central component to generate multiple complementary visualizations that enhance the standard t-SNE plot. **(a)** The full sensitivity Jacobian $\left(J_{z^{*}}\right)$ as a heatmap, showing the granular influence of input features on the embedding. **(b)** A feature influence map, created by summarizing the Jacobian column-wise to identify the most influential input features. **(c)** A sample sensitivity map, generated by summarizing the Jacobian row-wise and coloring points in the plot to highlight the most fragile or stable samples. **(d)** An uncertainty-aware visualization, which combines the Jacobian with input uncertainty to display the positional confidence of each point using hypothetical outcomes.

The computed Jacobian and output covariance matrix enable powerful visual enhancements that reveal the stability and interpretability of a t-SNE embedding. We propose three complementary visualization strategies, summarized in Figure 1.

#### 2.5.1 Visualizing feature influence

To visualize the influence of input features on the embedding, we utilize the sensitivity Jacobian matrix $J_{z^{*}}\in R^{2N\;\times \;ND}$ . For datasets with a manageable number of input dimensions and sample size (on the order of thousands when multiplied), the full Jacobian can be directly visualized as a heatmap (Figure 1a). The rows of this heatmap correspond to the embedding coordinates (e.g., the x and y coordinates for each sample), and the columns correspond to the input features. This provides a granular view of which specific inputs affect which specific output coordinates.

For larger datasets where the full Jacobian is too vast to interpret directly, we compute a summary. We calculate a total sensitivity score for each of the ND input features (Figure 1b). The score for input feature k , $s_{k}$ , is the sum of the absolute values of its derivatives across all 2N embedding coordinates:

$$s_{k}=\overset{2N}{\underset{i=1}{\sum }}|J_{z^{*},ik}|.$$ (12)

These scores quantify the overall influence of each input feature on the entire embedding. These scores can then be visualized as a heatmap. For structured data, such as a time-series with multiple genes, this summary vector can be reshaped into a matrix (e.g., timepoints × genes) to reveal systematic patterns of influence.

#### 2.5.2 Visualizing sample sensitivity

In addition to identifying influential features, we can visualize the intrinsic sensitivity of each individual sample’s position. This is achieved by summarizing the Jacobian row-wise (Figure 1c). First, for each of the 2N output coordinates, we compute a sensitivity score $r_{i}$ by summing the absolute values of the corresponding row’s elements:

$$r_{i}=\overset{ND}{\underset{k=1}{\sum }}|J_{z^{*},ik}|.$$ (13)

Since each sample j (for j=1,…,N ) in a 2D embedding is represented by two coordinates (an x - and y -coordinate, corresponding to rows 2j−1 and 2j of the Jacobian, respectively), we aggregate these scores to get a single, overall sensitivity score $S_{j}$ for that sample:

$$S_{j}=r_{2j-1}+r_{2j}=\overset{2j}{\underset{i=2j-1}{\sum }}\overset{ND}{\underset{k=1}{\sum }}|J_{z^{*},ik}|.$$ (14)

These per-sample scores $\left\{S_{1},\ldots ,S_{N}\right\}$ can then be used to color the points directly in the t-SNE plot using a sequential colormap. This immediately highlights which samples have the most stable versus the most fragile positions in the map.

#### 2.5.3 Visualizing positional uncertainty

To visualize the propagated positional uncertainty, which is captured by the output covariance matrix $\Sigma_{z}$ , we employ hypothetical outcome plots (Figure 1d). This technique involves drawing multiple random samples from the full multivariate Gaussian distribution $p\left(z*\right)\approx N\left(z*;\;z*\left(y*\right),\;\Sigma_{z}\right)$ . Each sample represents a plausible complete embedding given the input uncertainty. These outcomes can be rendered as a static overlay of semi-transparent points or, more powerfully, as a dynamic animation. However, displaying independent random samples as frames can result in a jerky, disconnected visual experience, making it difficult to perceive stable structures. To address this, we adopt a structured sampling approach to create a smooth animation, as detailed in our prior work on visualizing uncertainty in PCA (Zabel et al., 2023). This method traces a continuous path through a set of equiprobable embeddings, resulting in a smooth animation that greatly aids the visual perception of stable structures and correlated movements between points.

### 2.6 Implementation

The t-SNE cost function (Equations 1–4), the sensitivities (Equation 9), and the Gaussian error propagation terms (Equation 11) were implemented in Python using the JAX library (Bradbury et al., 2018) for its automatic differentiation and GPU acceleration capabilities. The Hessian and mixed-partial derivatives required by the IFT are computed automatically from a JAX implementation of the t-SNE cost function’s gradient. To handle the potentially large matrices involved, we employ matrix-free methods. The full output covariance matrix is constructed column-by-column using efficient Jacobian-vector products (JVPs) and vector-Jacobian products (VJPs) without explicitly instantiating the full mixed-Jacobian matrix. Further implementation details are provided in the Supplementary Method.

## 3 Results

We demonstrate the utility of our framework on two distinct and highly relevant bioinformatics use cases: first, a bulk multi-omics (RNA-seq and proteomics) time-series dataset with biological replicates to validate the full uncertainty propagation workflow, and second, a large-scale single-cell RNA-seq dataset to showcase the power of sensitivity analysis for feature attribution and cluster stability assessment.

### 3.1 Application to bulk RNA-Seq & proteomics: interpreting a metabolic switch in *Streptomyces coelicolor*

In our first use case we applied our framework to a bulk multi-omics (RNA-seq and proteomics) dataset from *Streptomyces coelicolor* (*S. coelicolor*) (Sulheim et al., 2020), a bacterium known for its complex metabolic shifts. We first analyzed a time-series gene expression dataset. The dataset consists of RNA-seq measurements at eight timepoints ( $t_{1}$ to $t_{8}$ ) during phosphate depletion, with three biological replicates per timepoint. Prior work (Sulheim et al., 2020) demonstrated that a major metabolic switch occurs between $t_{3}$ and $t_{4}$ in response to phosphate depletion. We focused our analysis on the top 5% most variable genes (396 genes, based on standard variance) to ensure matrices of appropriate size for visualization. The mean expression across replicates for each timepoint was used as the point estimate $Y*\in R^{8\times 396}$ , and the variance of these means, estimated from the replicates, formed the diagonal input covariance matrix $\Sigma_{y}$ .

**FIGURE 2:**
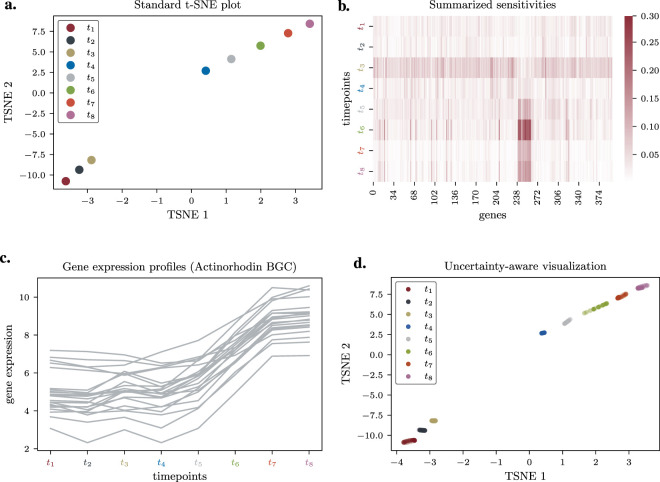
Sensitivity Analysis and Uncertainty Visualization of *S. coelicolor* Time-Series Data. **(a)** Standard t-SNE embedding of mean gene expression profiles across eight timepoints, showing separation between early and late phases. **(b)** Heatmap of summarized sensitivity scores, highlighting the total influence of each gene (column) at each timepoint (row). A block of high-sensitivity genes is evident at late timepoints. **(c)** Mean expression profiles (log-scale) of the genes identified as most influential in panel B, corresponding to the actinorhodin biosynthetic gene cluster (BGC). **(d)** Uncertainty-aware visualization showing hypothetical outcomes of the embedding based on propagated replicate variance. The position of timepoint $t_{6}$ is revealed to be less stable.

A standard t-SNE embedding of the mean expression data clearly separates the early $\left(t_{1}-t_{3}\right)$ from the subsequent $\left(t_{4}-t_{8}\right)$ timepoints, reflecting a known major metabolic switch (Figure 2a). While this visualization confirms the expected biology, it offers no further insight into what drives this separation or how stable it is.

We applied our framework (Equation 9) to compute the sensitivity Jacobian $J_{z*}=\frac{\partial z^{*}}{\partial y}$ for this embedding (see Supplementary Figure S1 for a heatmap visualization). To identify the most influential genes and timepoints, we calculated a total sensitivity score for each input feature by summing the absolute values of its derivatives across all 16 embedding coordinates (see Equation 12). This analysis revealed some interesting results: a specific subset of genes exhibited exceptionally high sensitivity scores, primarily at the later timepoints $\left(t_{5}-t_{8}\right)$ , with the highest sensitivity at $t_{6}$ (Figure 2b). A query of their gene annotations identified these as the biosynthetic gene cluster (BGC) for the antibiotic actinorhodin (SCO5071-SCO5091). Their expression profiles show a characteristic sharp upregulation precisely during these late timepoints (Figure 2c). This result directly and quantitatively links the primary visual feature of the t-SNE plot—the separation of the late timepoints—to a specific, critical biological process. Furthermore, the analysis highlighted that the entire embedding was highly sensitive to the expression profile of sample $t_{3}$ (see Figure 2b) underscoring the pivotal role of this transitional timepoint just prior to the metabolic switch.

Next, we used the Jacobian to propagate the input uncertainty derived from the biological replicates (see Equation 11). The resulting uncertainty visualization (Figure 2d; animated version available at https://github.com/Integrative-Transcriptomics/tsne/blob/main/paper/figures_and_animations/M145.gif) showed that most timepoints are positioned with high confidence. However, timepoint $t_{6}$ displayed notably larger positional uncertainty, with its position varying along the trajectory between the $\left(t_{4},t_{5}\right)$ and $\left(t_{7},t_{8}\right)$ sub-clusters. This ambiguity is biologically plausible, as $t_{6}$ represents a transitional metabolic state. Our framework not only visualizes this instability but, through the sensitivity analysis, attributes it to the variance in the expression of the highly influential actinorhodin BGC genes during this critical period.

To demonstrate the broad utility of our framework across different data modalities, we extended our analysis to a corresponding proteomics dataset from the same *S. coelicolor* time-series experiment (Sulheim et al., 2020). We applied our sensitivity analysis to the t-SNE embedding of the proteome data to identify the most influential proteins driving its structure.

The results provided a cross-modality validation of our findings. The sensitivity analysis of the proteome data independently highlighted proteins belonging to the very same metabolic pathway—the actinorhodin biosynthetic gene cluster—that was identified as the key driver in the transcriptomics data (see Supplementary Figure S2). This demonstrates that our framework can consistently pinpoint core biological drivers across distinct molecular layers, showcasing its robustness and utility for integrative multi-omics interpretation. The full t-SNE embedding, sensitivity analysis, uncertainty propagation, and detailed figures for the proteomics data are provided in the Supplementary Results, Section 2.2.

### 3.2 Application to single-cell RNA-Seq: assessing the stability and drivers of immune cell embeddings

To demonstrate our method’s scalability and utility on larger, contemporary datasets, we analyzed a single-cell transcriptomics dataset (GEO accessions: dataset GSE164378, sample GSM5008737) from Hao et al. (Hao et al., 2021). Data was further annotated by the corresponding metadata as well as cell type labels for six major cell types provided by Dietrich et al. (2024). The resulting dataset contained single cell expression data from six major immune cell types from eight human donors. After a standard preprocessing workflow (scanpy.pp.recipe_zheng17 (Zheng et al., 2017)) using Scanpy (Wolf et al., 2018), we retained the 100 most highly variable genes across 149,482 cells. We applied our framework in two complementary ways: first, we performed a per-cell sensitivity analysis on a subset of the data to identify fragile cell positions within the embedding, and second, we conducted an uncertainty propagation analysis on pseudo-bulk profiles to assess the stability of entire cell type clusters.

#### 3.2.1 Per-cell sensitivity analysis reveals unstable regions of the embedding

To first assess the intrinsic stability of individual cell positions in the t-SNE embedding, we performed a sensitivity analysis on the single-cell-resolution data. We computed a t-SNE embedding on a random subset of 1,000 cells and then calculated the full sensitivity Jacobian for this embedding according to Equation 9. The resulting Jacobian is a high-dimensional matrix detailing how each of the 100 genes influences the coordinates of every one of the 1,000 cells. While this full matrix can be inspected directly to identify the specific genes that a particular cell’s position is most sensitive to, our primary goal here was to create a high-level visual summary of overall cell stability.

**FIGURE 3:**
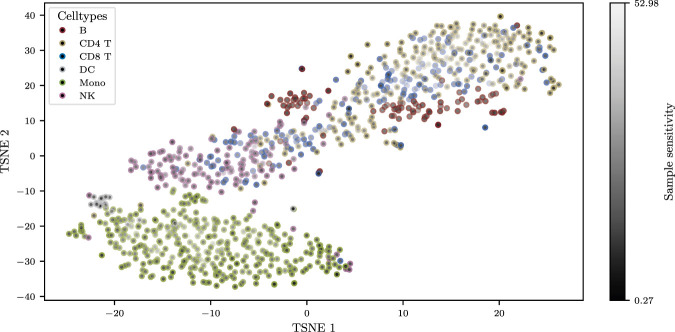
Per-Cell Sensitivity Analysis of a Single-Cell t-SNE Embedding. t-SNE embedding of 1,000 single cells. Cell type identity is indicated by the colored border of each point. The fill color represents the intrinsic sensitivity of each cell’s position, calculated from a row-wise summary of the Jacobian (black = low sensitivity, light gray = high sensitivity).

To achieve this, we summarized the Jacobian to derive a single sensitivity score for each cell (see Equations 13, 14) and used these scores to color the points in the t-SNE plot, with a black-to-gray gradient indicating low-to-high sensitivity and colored borders retaining cell type identity. The resulting t-SNE visualization (Figure 3) revealed a complex stability landscape. The most pronounced sensitivity was observed at the interface between the CD4 and CD8 T cell populations, precisely where the two cell types are not clearly resolved in the embedding. This immediately highlights a region of known biological similarity and visual ambiguity as being the most fragile part of the map. Furthermore, the analysis revealed a non-obvious pattern within the well-separated clusters. Cells located in the dense cores of clusters like the monocytes often exhibited higher sensitivity than cells at the sparser cluster peripheries.

This analysis demonstrates that our framework can be used as a powerful diagnostic tool directly on standard single-cell embeddings, providing a data-driven method to identify which cells or regions of the t-SNE map are most “fragile” and whose positions should be interpreted with caution, even in the absence of an explicit uncertainty model.

### 3.2.2 Cluster stability analysis using pseudo-bulk profiles

Next, to assess the stability of the overall cell type clusters, we created pseudo-bulk profiles by summarizing the expression data for each cell type within each donor, resulting in 6×8=48 distinct samples. For each sample, we computed the mean expression profile (y*) and the variance across all cells within that group $\left(\Sigma_{y}\right)$ , providing a direct measure of intra-celltype heterogeneity.

**FIGURE 4:**
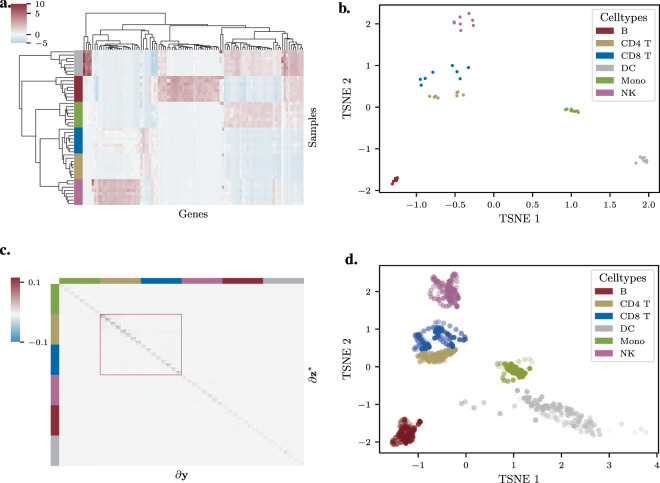
Sensitivity and Stability Analysis of Immune Cell Subsets. **(a)** Clustered heatmap of mean expression profiles for 48 pseudo-bulk samples (6 cell types x 8 donors). Samples cluster primarily by cell type, with CD4 and CD8 T cells showing high similarity. **(b)** Standard t-SNE embedding of the 48 samples. Major cell types form distinct clusters, while CD4 and CD8 T cells are closely co-located. **(c)** Heatmap of the sensitivity Jacobian. The strong diagonal indicates that each sample’s embedding is primarily sensitive to its own input features. CD4 and CD8 T cells exhibit the highest sensitivity scores. Note that cell type colors follow the color code shown in Figure 4d. **(d)** Uncertainty-aware visualization of the t-SNE embedding. Ellipses represent the propagated intra-population variance.

As expected, unsupervised clustering of the mean expression profiles shows that samples group primarily by cell type (Figure 4a). A standard t-SNE embedding of the 48 samples confirms this structure, revealing distinct clusters for B cells, NK cells, Monocytes, and Dendritic Cells (DCs) (Figure 4b). Due to their highly similar expression profiles, CD4 and CD8 T cells are positioned closely together and are not fully resolved into separate clusters, though they remain linearly separable.

Next, we applied our sensitivity analysis framework to this dataset. This revealed a notable structure in the Jacobian matrix: it was strongly diagonal (Figure 4c). This indicates that the position of a given sample’s embedding (e.g., Patient 1’s B cells) is almost exclusively sensitive to its own high-dimensional expression profile, with minimal influence from other samples. This suggests a relatively independent embedding for each sample. Crucially, the analysis also showed that the embeddings for CD4 and CD8 T cells were the most sensitive to input perturbations, consistent with their close proximity and the inherent difficulty in resolving them.

Finally, we propagated the cell-to-cell variance for each sample through the t-SNE map to visualize the stability of their positions (Figure 4d; animated version available at https://github.com/Integrative-Transcriptomics/tsne/blob/main/paper/figures_and_animations/hao_mean_animation.gif). This result illustrates a key insight from our framework: positional uncertainty in a t-SNE embedding is a product of both the mapping’s intrinsic sensitivity and the input data’s variance. For example, while the CD4 T cell embeddings have high sensitivity, their low intra-population variance (Supplementary Figure S3) results in a relatively stable and certain position. Conversely, the DC population, which exhibits higher expression heterogeneity (high input variance), results in a much more uncertain embedding position despite having lower intrinsic sensitivity than the T cells. This analysis demonstrates how our method can deconstruct the sources of uncertainty, allowing for a more nuanced interpretation of cluster stability in a t-SNE visualization.

## 4 Discussion

In this work, we have introduced a computational framework to move beyond static t-SNE plots, providing a principled methodology for both sensitivity analysis and uncertainty-aware visualization. Our central contribution is a method to efficiently compute the t-SNE Jacobian, which serves as a powerful diagnostic for interpretability and a foundation for robust uncertainty quantification. A key advantage of our approach, based on the Implicit Function Theorem, is that it operates directly on the optimality conditions of the t-SNE cost function. This makes our framework solver-agnostic: it can be applied to any converged t-SNE embedding, regardless of the specific optimization algorithm or software package used to generate it, from Barnes-Hut implementations (e.g., Van Der Maaten, 2014) to GPU-accelerated solvers (e.g., Chan et al., 2018).

While a standard t-SNE plot effectively visualizes high-dimensional structure, interpreting this structure requires a demanding mental leap back to the original features to assess their relevance. Our framework is designed to bridge this interpretational gap by embedding analytical insights directly into the visualization. Instead of forcing users to guess which genes define a cluster, our feature influence maps provide a direct, data-driven answer. Similarly, rather than subjectively assessing cluster tightness, our uncertainty visualizations offer a quantitative measure of positional stability. By offloading this analytical burden from the user to the computation, our visualizations allow users to interpret the stability t-SNE plots more easily.

Our analyses across two distinct and challenging biological data modalities underscore the versatility of this framework. On bulk multiomics data, the method not only validated its uncertainty propagation capabilities against true biological replicates but also performed successful feature attribution, directly linking the visual separation of a time-series to the expression dynamics of a key antibiotic-producing gene cluster. Our application to single-cell data demonstrated the framework’s dual utility as a multi-scale diagnostic tool. The per-cell sensitivity analysis revealed a complex stability landscape, highlighting not only the expected fragility at the unresolved boundary of T-cell subtypes but also the counter-intuitive finding that dense cluster cores can be more sensitive than their peripheries. Building on this, the pseudo-bulk analysis deconstructed the sources of overall cluster stability, showing how positional uncertainty is a product of both intrinsic sensitivity and biological heterogeneity.

Our work contributes to the broader effort within eXplainable AI (XAI) to bring interpretability to non-linear embeddings, a challenge being addressed from multiple angles. One major branch of research modifies the embedding algorithm itself. The Ut-SNEs preprint, for instance, integrates input uncertainty directly into the optimization objective to produce a single, uncertainty-aware embedding (Ma and Chen, 2024). In contrast, post-hoc frameworks, including our own, analyze a standard, converged embedding without altering the algorithm.

Traditional post-hoc approaches, adapted from linear methods, attempt to interpret embedding axes, for example, by creating curved biplots (Coimbra et al., 2016). Besides that, many of the post-hoc methods rely on approximation or stochastic sampling. A popular strategy involves training a surrogate model, such as using a random forest to explain UMAP clusters (Ehiro, 2023) or adapting LIME (Ribeiro et al., 2016) to explain local t-SNE neighborhoods (Bibal et al., 2020). While powerful, these methods are inherently approximate, as they explain the surrogate model, not the embedding algorithm itself. Other post-hoc approaches assess reliability through resampling, for example, using bootstrapping to test the structural stability of clusters (Kobak and Berens, 2019), or by evaluating projection fidelity to quantify how well the 2D map represents true high-dimensional neighborhoods (Han et al., 2022). Our framework defines a distinct class of post-hoc analysis that is fully analytical and deterministic, moving beyond these approximate and stochastic techniques.

Our framework aligns with and extends the most recent class of methods: gradient-based explanation. The concurrent work of Corbugy et al. (2024) also uses the Implicit Function Theorem to provide instance-specific explanations. The fundamental difference lies in the problem formulation. Their method explains the position of a single instance under the simplifying assumption that all other embedded points are held constant. In contrast, our framework addresses the complete, simultaneous optimization problem, computing the full Jacobian $\frac{\partial z}{\partial y}$ . This more challenging approach yields a richer result, allowing us to analyze the coupled dynamics of the entire visual structure. Moreover, our primary application of this full Jacobian—propagating input uncertainty to generate uncertainty-aware visualizations—is a distinct contribution not explored in these other explanation-focused works.

It is also important to situate our definition of stability. Much of the existing literature on t-SNE stability focuses on the “global” variability arising from different random initializations or hyperparameter choices (Belkina et al., 2019). Our work is complementary, focusing instead on the “local” stability of a single, converged embedding, which is crucial for interpreting the final plot presented in a study. Our approach here extends the core ideas of uncertainty propagation we previously developed for PCA (Zabel et al., 2023) to the more complex, iterative optimization setting of t-SNE.

Despite its utility, our method has limitations that suggest avenues for future research. The primary constraint is computational complexity. Our empirical benchmarks (see Supplementary Section 2.4) confirm that the cost of computing the output covariance scales approximately cubically with the number of samples $\left(O\left(N^{3}\right)\right)$ , driven by the Hessian pseudoinversion, and roughly linearly with the number of features (O(D)) . This computational overhead is substantial compared to a standard t-SNE run, making our full uncertainty propagation demanding for datasets with a very large number of samples (e.g., N>10,000 ). However, the analysis also demonstrates that the framework remains highly practical ( < 1 h runtime on a typical server) for the moderate sample sizes common in many applications, such as the pseudo-bulk and proteomics analyses presented in this paper. For larger datasets, that are typical for example, for single-cell experiments, future work could therefore explore scalable approximations, such as iterative solvers for the pseudoinverse-vector products, to mitigate this bottleneck. Second, our uncertainty propagation relies on a first-order Taylor approximation (Equation 10). The core quantitative output of our method is the full output covariance matrix, $\Sigma_{z}$ , which provides a complete diagnostic of the embedding’s variance and covariance structure. Our visualizations, such as hypothetical outcome plots, are principled renderings of this matrix, designed to intuitively convey correlated uncertainties that are lost when only visualizing marginal confidence ellipses. However, the accuracy of this underlying covariance matrix is dependent on the local linearity of the t-SNE mapping. Lastly, our analysis is local to a single t-SNE optimum. For a comprehensive stability assessment, we therefore suggest a two-stage workflow that combines our local analysis with methods for assessing global stability. An analyst would first use established techniques, such as running t-SNE from multiple random initializations (Belkina et al., 2019), to select a globally stable and representative embedding. Our framework would then be applied to this single, chosen map to probe its local stability, revealing which structures are robust to input data noise and identifying their feature drivers.

Finally, while this work has focused on t-SNE, the underlying IFT-based framework is not inherently restricted to this one algorithm. We chose t-SNE as the initial application for this work due to its foundational status in the field and its well-defined, continuously differentiable cost function. In principle, however, our method can be extended to any embedding technique that relies on minimizing a differentiable objective function. The most logical and impactful next step would be Uniform Manifold Approximation and Projection (UMAP) (McInnes et al., 2018), another cornerstone of modern data visualization. Extending our work to UMAP would be an important step towards a unified framework for assessing the stability of non-linear embeddings.

In conclusion, this work provides a practical and powerful toolkit for enhancing the rigor and interpretability of t-SNE visualizations, and thus creates a more effective and insightful visual representations of the embedded data. By enabling researchers to quantify the influence of their features and visualize the uncertainty in their embeddings, our framework promotes a more critical and nuanced understanding of high-dimensional data. As AI-driven visualization becomes more central to biological discovery, integrating such diagnostics for trust and transparency will be essential.

## Data Availability

The original contributions presented in the study are included in the article/Supplementary Material, further inquiries can be directed to the corresponding authors.
