# Supplementary material for: Visualizing stability: a sensitivity analysis framework for t-SNE embeddings
Source: Front Bioinform. 2026 Jan 2;5:1719516. doi: 10.3389/fbinf.2025.1719516 (PMC12808344; doi:10.3389/fbinf.2025.1719516)
Supplement: Supplementary file 1 [file DataSheet1.pdf]

Supplementary Material

1 SUPPLEMENTARY METHODS

1.1 Efficient Computation of the Output Covariance Matrix

The explicit formation of the Jacobian $\boldsymbol{J}_{\boldsymbol{z}^*} = \frac{\partial \boldsymbol{z}^*}{\partial \boldsymbol{y}}$ and the input covariance $\boldsymbol{\Sigma}_y$ can be computationally prohibitive. We therefore compute the output covariance matrix $\boldsymbol{\Sigma}_z = \boldsymbol{J}_{\boldsymbol{z}^*}\boldsymbol{\Sigma}_y(\boldsymbol{J}_{\boldsymbol{z}^*})^\intercal$ column by column using a sequence of matrix-free operations. The k-th column of $\boldsymbol{\Sigma}_z$ is given by $\boldsymbol{\Sigma}_z \boldsymbol{e}_k$, where $\boldsymbol{e}_k$ is the k-th standard basis vector. Using the IFT result from the main text (Eq. (9)), this product can be efficiently calculated from right to left via a series of Jacobian-vector products (JVPs) and vector-Jacobian products (VJPs), as implemented in frameworks like JAX:

1. $\boldsymbol{v}_a = ((\boldsymbol{H}_{\boldsymbol{zz}})^+)^\intercal \boldsymbol{e}_k$
2. $\boldsymbol{v}_b = (\frac{\partial^2 C}{\partial \boldsymbol{y} \partial \boldsymbol{z}})^\intercal \boldsymbol{v}_a$ (Vector-Jacobian product)
3. $\boldsymbol{v}_c = \boldsymbol{\Sigma}_y \boldsymbol{v}_b$ (Covariance-vector product)
4. $\boldsymbol{v}_d = \frac{\partial^2 C}{\partial \boldsymbol{y} \partial \boldsymbol{z}} \boldsymbol{v}_c$ (Jacobian-vector product)
5. $\boldsymbol{\Sigma}_z[:, k] = (\boldsymbol{H}_{\boldsymbol{zz}})^+ \boldsymbol{v}_d$

This procedure avoids instantiating the full mixed-partial derivative matrix, significantly reducing memory requirements.

2 SUPPLEMENTARY RESULTS

2.1 Full Sensitivity Jacobian for Bulk RNA-Seq Data

The summarized sensitivity scores in the main text (Fig. 2B) were derived from the full Jacobian matrix $\boldsymbol{J}_{\boldsymbol{z}^*} \in \mathbb{R}^{16 \times 3168}$, shown in Figure S1. The rows correspond to the $8 \times 2 = 16$ embedding coordinates, and the columns correspond to the $8 \times 396 = 3168$ input gene expression features. The figure highlights that sensitivities are not uniformly distributed, with specific embedding coordinates being highly sensitive to particular subsets of genes at specific timepoints. One such highly sensitive subset of genes at time point t_6 corresponds to the actinorhodin biosynthetic gene cluster.

2.2 Proteomics Analysis of the *S. coelicolor* Time-Series

To demonstrate the applicability of our framework to diverse omics data, we performed a parallel analysis on a proteomics dataset. This dataset, corresponding to the same time-series experiment as the transcriptomics data in the main text, provides an opportunity for cross-modality validation. The dataset consists of protein abundance measurements for 3342 proteins across the same eight timepoints. Similar to the RNA-seq analysis, we focused on the 5 % most variant proteins (in terms of the standard variance) and used the mean abundance across replicates as the input for the t-SNE embedding.

Supplementary Figure S2 details the results of this analysis. The t-SNE embedding of the proteome data (Supp. Fig. S2A) shows a clear temporal progression, separating early ($t_1 - t_3$) and late ($t_4 - t_8$) timepoints in a manner consistent with the transcriptomics data, confirming that the major metabolic switch is also evident at the protein level.

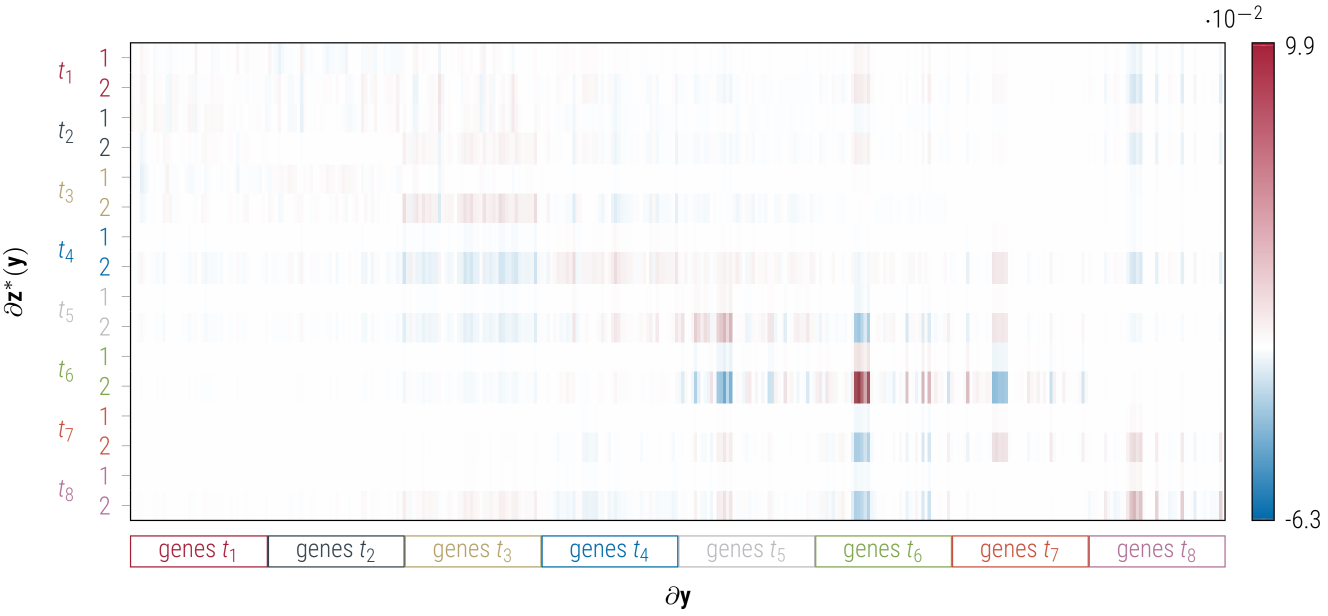

Figure S1: **Full Sensitivity Jacobian for the *S. coelicolor* bulk RNA-seq t-SNE Embedding.** Heatmap of the complete Jacobian matrix $\boldsymbol{J}_{\boldsymbol{z}^*}$.

We then applied our sensitivity analysis framework to identify the most influential proteins driving this structure. The summarized sensitivity heatmap (Supp. Fig. S2B) reveals that a distinct set of proteins exhibit high sensitivity scores, particularly in the later timepoints. Upon annotation, these high-sensitivity proteins were identified as key enzymes within the actinorhodin biosynthetic gene cluster, which show an elevated protein expression at timepoints $t_5 - t_8$ as a response to the induced metabolic switch (Supp. Fig. S2C).

Next, we used the Jacobian to propagate the input uncertainty derived from the biological replicates. The resulting uncertainty visualization (see Fig. S2D) showed that most timepoints are positioned with high confidence. However, the visualization revealed (particularly evident in the corresponding animation `https://github.com/Integrative-Transcriptomics/tsne/blob/main/paper/figures_and_animations/M145_proteomics.gif`), that timepoint t_6 appears to dynamically switch its proximity between the preceding timepoints (t_4, t_5) and the subsequent ones (t_7, t_8). This ambiguity is biologically plausible, as t_6 represents a transitional metabolic state. Our framework not only visualizes this instability but, through the sensitivity analysis, attributes it to the variance in the expression of the highly influential actinorhodin BGC genes during this critical period, a result that also was found on the transcriptomic level.

2.3 Input Variance for Single-Cell RNA-Seq Analysis

The analysis in the main manuscript revealed that the positional uncertainty of a sample in the t-SNE embedding is a product of both the embedding's intrinsic sensitivity and the input data's variance. To provide the underlying data for this conclusion in our single-cell RNA-seq analysis, we here visualize the input variance that was propagated through the t-SNE map.

Supplementary Figure S3 displays a clustered heatmap of the expression variances calculated for each of the 48 pseudo-bulk samples. This heatmap visually confirms that the pseudo-bulk profiles for Dendritic Cells (DCs) exhibit substantially higher expression variance across a range of genes compared to the

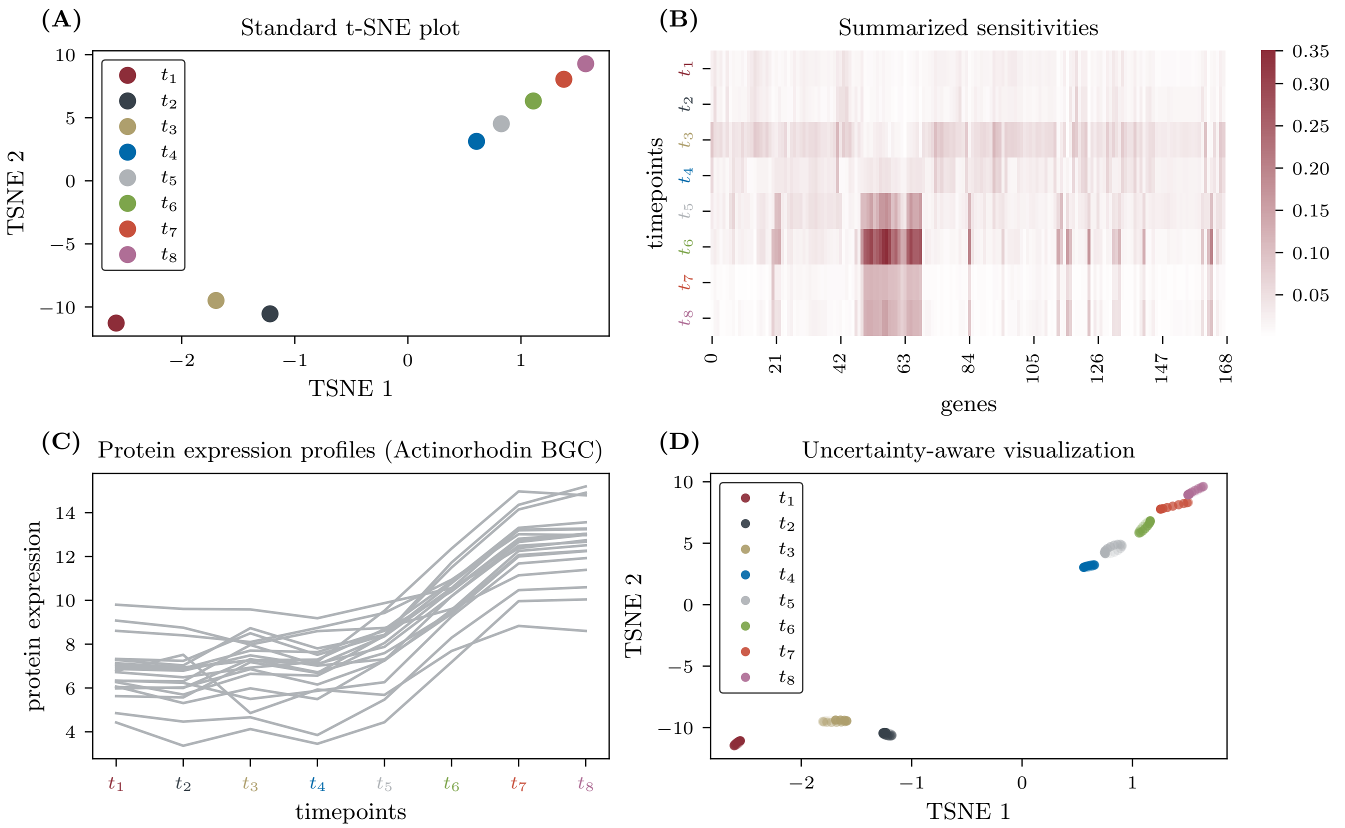

Figure S2: **Sensitivity Analysis and Uncertainty Visualization of *S. coelicolor* Time-Series Proteome Data.** **(A)** Standard t-SNE embedding of mean protein expression profiles across eight timepoints, showing separation between early and late phases. **(B)** Heatmap of summarized sensitivity scores, highlighting the total influence of each protein (column) at each timepoint (row). A block of high-sensitivity proteins is evident at late timepoints. **(C)** Mean expression profiles (log-scale) of the proteins identified as most influential in panel B, corresponding to the actinorhodin biosynthetic gene cluster (BGC). **(D)** Uncertainty-aware visualization showing hypothetical outcomes of the embedding based on propagated replicate variance.

other, more transcriptionally homogeneous cell types. This high input variance, combined with a moderate embedding sensitivity, is the direct cause of the larger positional uncertainty for the DC cluster observed in the main manuscript (Main Text, Fig. 4D).

2.4 Runtime and Scalability Analysis

To empirically validate the computational complexity of our framework and clarify its practical scalability, we performed a series of benchmarks on randomly generated datasets. We varied both the number of samples (N) and the number of feature dimensions (D) and measured the wall-clock time for both a standard t-SNE forward pass and for the subsequent computation of the full output covariance matrix using our method.

The results, shown in Supplementary Figure S4, confirm the theoretical complexity discussed in the main text. The runtime for covariance computation is largely dominated by the number of samples N. As shown in Supplementary Figure S4A, the scaling is consistent with the $O(N^3)$ complexity of the Hessian pseudoinversion step. In contrast, the scaling with respect to the number of features D appears roughly linear (Supplementary Figure S4B), and is therefore much more favorable,

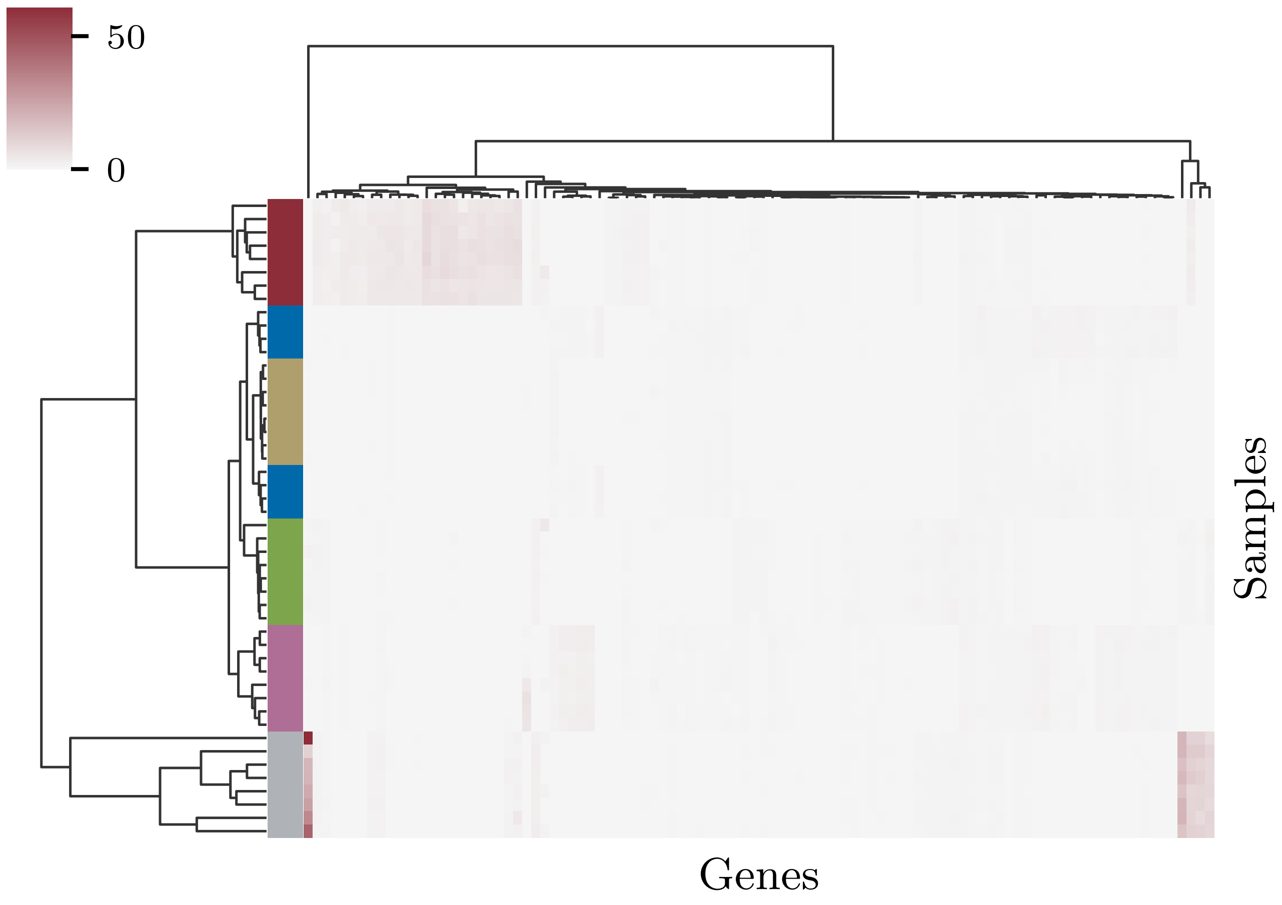

Figure S3: **Input Expression Variance for Pseudo-Bulk PBMC Samples.** Clustered heatmap of the input expression variances used for uncertainty propagation in the single-cell analysis. Rows correspond to the 48 pseudo-bulk samples (6 cell types x 8 donors), and columns correspond to the 100 most variable genes. Dendritic Cells (DCs, gray) exhibit notably higher expression variance across several genes compared to other cell types. This high input variance is the primary driver of the larger positional uncertainty observed for the DC cluster in the final t-SNE visualization (Main Text, Fig. 4D). Row colors for cell types correspond to those used in the main manuscript figures.

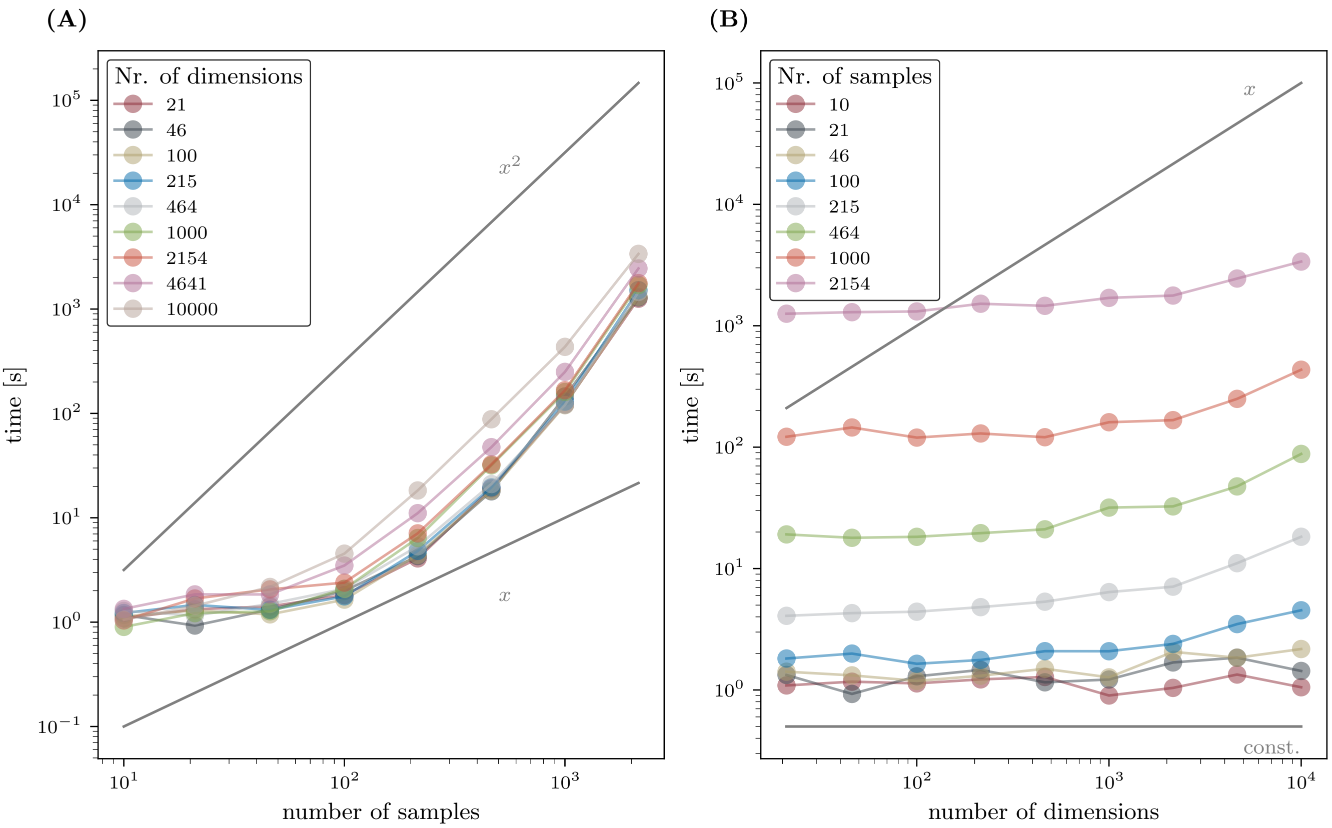

Figure S4: **Empirical Runtime and Scalability of the Proposed Framework.** All benchmarks were performed on an Intel Xeon CPU E5-2698 v3 @ 2.30 GHz (64 cores) and 378 GB of memory. **(A)** Runtime for covariance computation as a function of the number of samples (N), for several fixed feature dimensions (D). **(B)** Runtime for covariance computation as a function of the number of features (D), for several fixed sample sizes (N). The scaling with respect to the number of features is approximately linear.
